# The Prognostic Determinant of Interleukin-10 in Patients with Acute Ischemic Stroke: An Analysis from the Perspective of Disease Management

**DOI:** 10.1155/2021/6423244

**Published:** 2021-07-17

**Authors:** Wen Sun, Shuhui Wang, Shanji Nan

**Affiliations:** ^1^School of Public Health, Weifang Medical University, Weifang, China; ^2^Department of Clinical Laboratory, Qingdao Central Hospital, Qingdao, China; ^3^Department of Neurology, Second Hospital of Jilin University, Changchun, China

## Abstract

**Background:**

In patients with ischemic stroke, the role of anti-inflammatory cytokine Interleukin-10 (IL-10) in predicting risk and outcomes is not very clear. This study is aimed at prospectively assessing the prognostic determinant value of IL-10 in patients with acute ischemic stroke in a cohort of Chinese people.

**Methods:**

In a prospective cohort study, consecutive first-ever patients with acute ischemic stroke admitted to our hospital were included from October 2019 to October 2020. The serum level of IL-10 was measured at baseline. A structured follow-up telephone interview was performed on day 90 after admission. Logistic regression analyses were used to assess the prognostic value of IL-10 to predict the poor functional outcome (defined as a modified Rankin Scale score of 3 to 6) and mortality.

**Results:**

The median age of the 236 enrolled patients was 65 years (interquartile range (IQR), 56-76), and 57.6% were male. There was a negative correlation between the National Institutes of Health Stroke Scale (NIHSS) score and IL-10 serum levels (*r* (Spearman) = −0.221, *P* = 0.001). Patients with elevated IL-10 levels (> the highest quartile = 5.24 pg/mL; *n* = 79) were at significantly lower risk of poor functional outcomes (odds ratio (OR), 0.35; 95% confidence interval (CI), 0.19 to 0.63; *P* < 0.001) and mortality (OR = 0.24; 95% CI = 0.11–0.52; *P* < 0.001) compared with patients with IL-10 levels in the lowest three quartiles.

**Conclusions:**

Reduced serum levels of IL-10 were independently associated with both the clinical severity at admission and a poor functional prognosis in ischemic stroke patients, suggesting that the anti-inflammatory cytokine IL-10 was an important prognostic determinant.

## 1. Introduction

Acute ischemic stroke is one of the main causes of death and the leading cause of disability in adults worldwide [1, 2]. In China, ischemic stroke is a common disorder with almost 2 million new or recurrent events per year [3], and one study indicated annual estimates of 11 million prevalent cases of stroke in 2013 [4]. Stroke leads to the highest disability-adjusted life-year loss of any disease in China [3]. In the past ten years, China has made outstanding achievements in stroke prevention and control [5]. However, with the increase of population aging and the poor management of chronic risk factors, future stroke prevention and treatment work still face challenges [3].

Inflammation and oxidative stress pathways are the pathophysiological mechanisms involved during ischemic stroke [6]. Global brain inflammation after a stroke might continuously affect the patients' long-term functional outcomes [7]. Inflammation is a double-edged sword at particular stages after a stroke, which could be both detrimental and beneficial [8]. Various proinflammatory and anti-inflammatory responses after ischemic stroke are potential therapeutic strategies [9]. The mechanisms between systemic inflammation and poor stroke outcome had been proposed [10].

Elevated blood levels of inflammatory cytokines such as C-reactive protein (CRP) [11], Interleukin-6 (IL-6) [12, 13], IL-18 [14], IL-12 [15], tumor necrosis factor-*α* [16], and C–C chemokine ligand 2 (CCL-2) [17] were associated with poor outcome and increased mortality after stroke. Anti-inflammatory cytokines could inhibit the proinflammatory cytokine production and might be used for stroke treatment. Vila et al. [18] showed that a reduced level of IL-10 (an anti-inflammatory cytokine) was associated with neurological worsening in acute ischemic stroke. Similarly, another study also reported that reduced IL-10 concentrations were associated with a degree of neurological deficit and poor stroke outcome [19]. Interestingly, not all the literature conclusions are very consistent. One study showed that higher IL-10 levels were associated with poor outcomes in female patients with ischemic stroke [20], while another study reported that more elevated serum IL-10 was an independent prognosticator of ischemic stroke outcome [21]. In addition, the prognostic value of IL-10 in ischemic stroke patients in another study was not confirmed [22].

The prognostic value of IL-10 in patients after acute ischemic stroke is not well documented. Further research is recommended to explain and unify these differences. Thus, this study is aimed at prospectively assessing the prognostic determinant value of anti-inflammatory cytokine IL-10 in patients with acute ischemic stroke in a cohort of Chinese people.

## 2. Subjects and Methods

### 2.1. Patients and Study Design

A single-center prospective cohort study was performed from October 1, 2019, to October 30, 2020. All patients with acute ischemic stroke diagnosed as the first onset were consecutively enrolled in our hospital during this period. The enrolled patients also needed to meet the following criteria: the diagnosis met the criteria of the World Health Organization [23] and was confirmed by Magnetic Resonance Imaging (MRI), with symptom onset within 3 days. Patients with (1) cerebral hemorrhage, (2) malignant tumor, (3) renal insufficiency, (4) surgery or trauma < 3 months, and (5) autoimmune diseases would be excluded. In addition, those patients without informed consent or fasting blood samples also would be excluded.

### 2.2. Clinical Variables

Within 24 hours of hospitalization, we collect primary patient data, including sex, age, residence (urban and rural), ethnicity, education, and marital status, medical insurance information, vascular risk factors (smoking status (nonsmokers, past smokers, and current smokers), consumption of alcohol, family history of stroke, history of transient ischemic attack (TIA), hypertension, diabetes, hyperlipidemia, and atrial fibrillation), therapies before admission (antihypertensive, hypoglycemic, anticoagulant, and statins), and acute treatment (IV thrombolysis and/or mechanical thrombectomy). We also record the following measurement information: height and weight (body mass index (BMI) was calculated based on height and weight, and BMI ≥ 28 kg/m^2^ was defined as obesity), arterial pressure (systolic and diastolic), body temperature, stroke severity at admission (assessed by the National Institutes of Health Stroke Scale (NIHSS) score (0-42, the higher the score, the more serious the disease)) [24], and lesion size (brain MRI was performed within 48 hours after admission to verify the stroke diagnosis; the infarct volume was calculated by using the formula 0.5 × *a* × *b* × *c* according to the diffusion-weighted imaging (DWI) sequences) [25]. Trial of ORG 10172 in Acute Stroke Treatment (TOAST) classification was used to classified ischemic strokes, which distinguishes large-vessel occlusive, small-vessel occlusive, cardioembolic, other, and unknown subtypes [26].

#### 2.2.1. Blood Sampling and Follow-Up

Fasting serum samples were routinely collected within the first 24 h after admission. Those samples were stored at –80°C before being tested for biomarkers. At discharge, length, cost, and treatment information during hospitalization were recorded. A structured follow-up telephone interview with the patient or the closest relative was performed on day 90 after admission. The patient's survival information was recorded. The functional outcome of surviving patients was assessed by the modified Rankin Scale (mRS) score [27], blinded to clinical data and laboratory tests. Patients were classified according to the mRS scores: poor functional outcome (defined as an mRS score of 3 to 6 points) and good outcome (defined as an mRS score of 0 to 2 points) [28].

### 2.3. Laboratory Testing

The measurement of the serum level of IL-10 was performed by the enzyme-linked immunosorbent assay (ELISA) sandwich method. The Human IL-10 ELISA Kit (No. ab100549) was used (Abcam plc., Shanghai, 201203, China). In this study, the testing sensitivity was 1.00 pg/mL, and the testing range was from 1.00 pg/mL to 100.00 pg/mL. The precision of intra-assay and interassay was 8.0% and 9.5%, respectively. The serum level of IL-6 was also tested by the Human IL-6 ELISA Kit (No. ab178013). In addition, serum levels of CRP, glucose, triglycerides, total cholesterol, high-density lipoprotein (HDL), and LDL (low-density lipoprotein) were also tested in our laboratory department.

### 2.4. Statistical Analysis

The study results were shown as the number (percentage) for categorical variables and the median (interquartile range (IQR)) for continuous variables. Correlation analysis between IL-10 and different variables was assessed by Spearman's rank correlation. The Mann–Whitney *U* test for continuous variables and chi-squared test for categorical variables were used to compare differences between groups. Stroke clinical severity at admission was dichotomized as minor (NIHSS, 0-5), moderate (NIHSS, 6-11), and severe (NIHSS ≥ 12) [29].

The prognostic determinant value of IL-10 for stoke functional outcome and mortality was assessed, and the odds ratios (OR) and 95% confidence intervals (CI) of the highest quartile IL-10 level (vs. lowest three quartiles) as compared with other risk factors were calculated by univariate and multivariate logistic regression analyses. In multivariate analysis, we included age, sex, obesity, vascular risk factors, therapies before admission, acute stroke treatment, stroke subtype, NIHSS at admission, infarct size, and serum levels of IL-6, Hs-CRP, and glucose.

Receiver operating characteristic (ROC) curve analysis was used to assess the cut-off value of IL-10 serum level as an indicator for screening of poor outcome and mortality, and results were reported as the area under the curve (AUC) and 95% CI. Diagnostic sensitivity and specificity also would be presented. Statistical software SPSS 24.0 (SPSS Inc., Chicago, IL, USA) was used for data statistics, and *P* values less than 0.05 (two-tailed) were considered to indicate significance.

### 2.5. Ethics Approval

The Ethics Committee of the First Affiliated Hospital of Jilin University reviewed and approved the research protocol (2019-08-007). Before entering the group, patients understood the research protocol, rights, and obligations and signed written informed consent. For patients who could not provide signatures, family members' signatures were also acceptable.

## 3. Results

### 3.1. Patients

**Figure 1:**
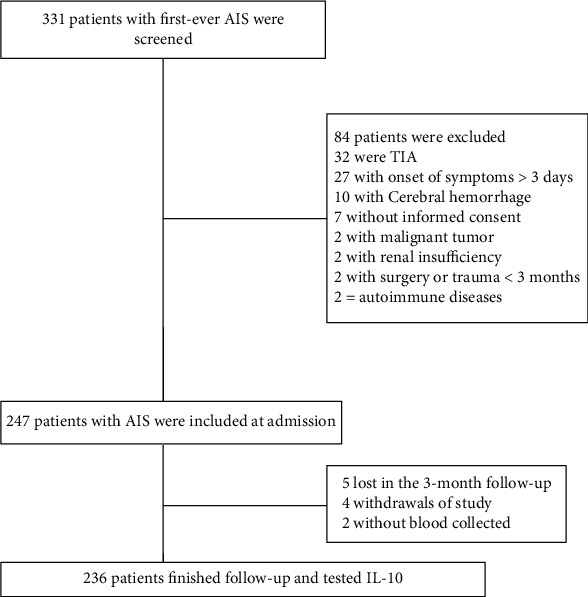
Research flow chart.

In the beginning, 331 patients with suspected first-ever ischemic stroke entered the scope of our study; 247 patients with acute ischemic stroke were included at admission. Finally, 236 patients with collected blood samples finished follow-up and tested IL-10 (Figure 1). The basic information of the enrolled patients was comparable to that of all screened patients (age (*P* = 0.08), sex (*P* = 0.55), and BMI (*P* = 0.21)).

### 3.2. Descriptive Characteristics of Included Patients

The median age of the enrolled patients was 65 years (IQR, 56-76), and 57.6% (*n* = 136) of the patients were male. Most of the enrolled patients were Han (95.3%) and enjoyed medical insurance (96.6%). The most common vascular risk factors were hypertension (74.2%), diabetes (28.8%), and hyperlipidemia (26.7%). In addition, a history of atrial fibrillation (10.2%), TIA (11.9%), and obesity (11.4%) were also uncommon. At admission, the NIHSS score and the median infarct size were 7 (IQR, 3–11) points and 15.8 (7.2-28.7) mL, respectively. More than one in ten patients (13.3%) received acute mechanical thrombectomy and/or IV thrombolysis therapy during hospitalization. At discharge, the median mRS score was 2 (IQR, 0-3), and the median length of hospitalization and hospitalization costs were 12 (9-17) days and 9988 (8315-14153) CNY, respectively. More information is presented in Table 1.

### 3.3. Main Results

**Figure 2:**
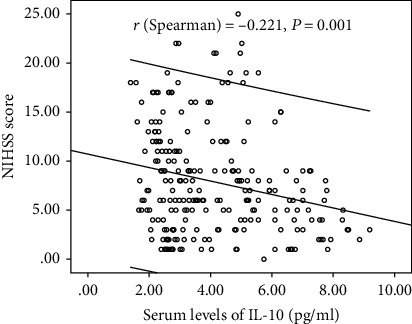
The correlation between NIHSS score and IL-10 serum levels. NIHSS: National Institutes of Health Stroke Scale; IL-10: Interleukin-10.

**Figure 3:**
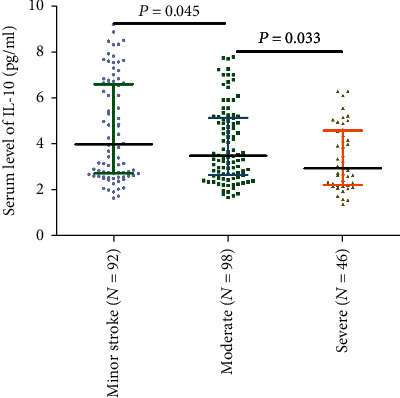
Serum IL-10 levels in groups stratified by stroke severity. All data are medians (IQR). *P* values refer to Mann–Whitney *U* tests. IL-10: Interleukin-10; IQR: interquartile ranges.

Serum IL-10 levels decreased with the increasing severity of stroke (evaluation by NIHSS score). As shown in Figure 2, there was a negative correlation between NIHSS score and IL-10 serum levels (*r* (Spearman) = −0.221, *P* = 0.001). Furthermore, serum IL-10 levels in minor stroke patients with an NIHSS score of 0 to 5 points (*N* = 92) were 4.00 (IQR, 2.72-6.59) pg/mL, in moderate patients with an NIHSS score of 6 to 11 (*n* = 98) were 3.49 (IQR, 2.65–5.14) pg/mL, and in severe patients with an NIHSS score greater than 11 (*n* = 46) were 2.95 (IQR, 2.23-4.59) pg/mL (Figure 3). As shown in Table 2, negative correlations between IL-10 serum levels and IL-6 (*P* = 0.012) and infarct size (*P* = 0.033) were also reported, unlike all others assessed.

### 3.4. IL-10 and Functional Outcome after 3 Months

**Figure 4:**
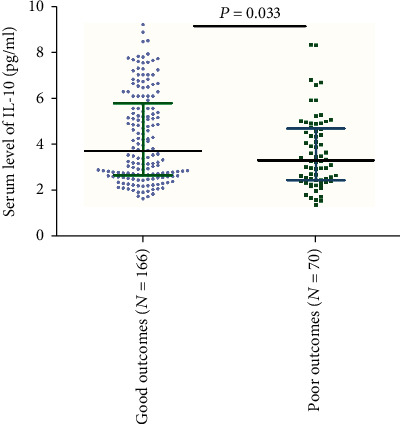
Serum IL-10 levels in groups stratified by the functional outcome. Poor functional outcome was defined as an mRS score of 3 to 6 points, and good outcome was defined as an mRS score of 0 to 2 points. All data are medians (IQR). *P* values refer to Mann–Whitney *U* tests. IL-10: Interleukin-10; IQR: interquartile ranges; mRS: modified Rankin Scale.

At follow-up, 70 patients (29.7%) had a poor functional outcome (mRS > 2). In these patients, the median IL-10 serum level was lower than in those patients with a good outcome (3.31 (IQR, 2.46-4.70) vs. 3.72 (IQR, 2.6-5.79); *P* = 0.033) (Figure 4). The ORs of the highest quartile IL-10 level (vs. lowest three quartiles) as compared with other risk factors were calculated by univariate and multivariate logistic regression analyses. With an unadjusted OR of 0.20 (95% CI, 0.09–0.42), IL-10 had a strong association with functional outcomes. In multivariable models adjusted for age, sex, obesity, vascular risk factors, therapies before admission, acute stroke treatment, stroke subtype, NIHSS at admission, infarct size, and serum levels of IL-6, Hs-CRP, and glucose, IL-10 levels in the highest quartile (>5.24 pg/mL) were associated with a reduced risk of a poor functional outcome (OR = 0.35; 95%CI = 0.19–0.63; *P* < 0.001). Conversely, the IL-6 serum level was positively associated with poor outcome (OR = 1.13; 95%CI = 1.04–1.21; *P* = 0.003) (Table 3).

**Figure 5:**
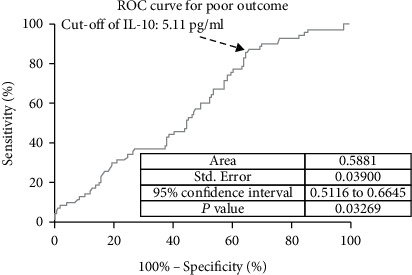
Receiver operating characteristic curve analysis was used to assess the value of IL-10 serum level as an indicator for screening of poor outcomes. Poor functional outcome was defined as an mRS score of 3 to 6 points. IL-10: Interleukin-10; mRS: modified Rankin Scale.

In ROC curve analysis, we calculated the cut-off value of IL-10 serum level as an indicator for screening of poor outcome, as presented in Figure 5. The critical value was 5.11 pg/mL with an AUC of 0.59 (95% CI, 0.51–0.66), showing a sensitivity of 87.14% and a specificity of 34.34%. With an AUC of 0.59, IL-10 was superior to CRP (AUC, 0.55; 95% CI, 0.50–0.59; *P* = 0.001) and white blood cell count (AUC, 0.53; 95% CI, 0.48-0.59; *P* < 0.001) and was within the range of the IL-6 (AUC, 0.61; 95% CI, 0.54-0.69; *P* = 0.103).

### 3.5. IL-10 and Death after 3 Months

**Figure 6:**
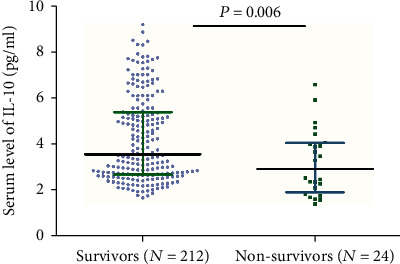
Serum IL-10 levels in groups stratified by survival state. All data are medians (IQR). *P* values refer to Mann–Whitney *U* tests. IL-10: Interleukin-10.

Twenty-four patients died during the follow-up. In these patients, the median IL-10 serum level was lower than in those surviving patients (2.91 (IQR, 1.90-4.05) vs. 3.55 (IQR, 2.67-5.37); *P* = 0.006) (Figure 6). The ORs of the highest quartile IL-10 level (vs. lowest three quartiles) as compared with other risk factors were also calculated by univariate and multivariate logistic regression analyses. With an unadjusted OR of 0.14 (95% CI, 0.06–0.30), IL-10 had a strong association with mortality. In multivariable models adjusted for age, sex, obesity, vascular risk factors, therapies before admission, acute stroke treatment, stroke subtype, NIHSS at admission, infarct size, and serum levels of IL-6, Hs-CRP, and glucose, IL-10 levels in the highest quartile (>5.24 pg/mL) were associated with a reduced risk of mortality (OR = 0.24; 95%CI = 0.11–0.52; *P* < 0.001). Conversely, the IL-6 serum level was positively associated with poor outcome (OR = 1.16; 95%CI = 1.02–1.25; *P* = 0.002) (Table 3).

**Figure 7:**
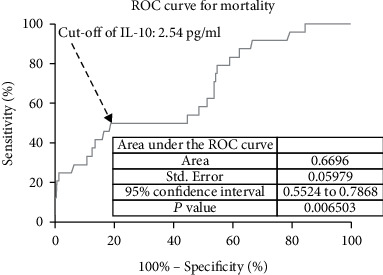
Receiver operating characteristic curve analysis was used to assess the value of IL-10 serum level as an indicator for screening of mortality. IL-10: Interleukin-10.

In ROC curve analysis, we calculated the cut-off value of the IL-10 serum level as an indicator for screening of death events as presented in Figure 7. The critical value was 2.54 pg/mL with an AUC of 0.67 (95% CI, 0.55–0.79), showing a sensitivity of 50.00% and a specificity of 81.13%. With an AUC of 0.67, IL-10 was superior to CRP (AUC, 0.61; 95% CI, 0.54–0.69; *P* < 0.001) and white blood cell count (AUC, 0.57; 95% CI, 0.50-0.68; *P* < 0.001) and was within the range of the IL-6 (AUC, 0.69; 95% CI, 0.60-0.81; *P* = 0.075).

## 4. Discussion

IL-10, a quintessential immunosuppressive anti-inflammatory cytokine, can resolve inflammation and promote wound repair at peripheral sites. In this prospective, cohort study, we found that low serum levels of IL-10 were independently associated with both the clinical severity at admission and a poor functional prognosis in ischemic stroke patients, suggesting that the anti-inflammatory cytokine IL-10 was an important prognostic determinant.

In patients with ischemic stroke, the role of IL-10 in predicting risk and outcomes is not very clear [30]. In the limited number of clinical studies, higher IL-10 levels seen postictus are related to worse outcomes [20, 21]. However, in this study, we showed that elevated IL-10 was associated with a more favorable prognosis, which had been supported by two previous studies [18, 19]. Similarly, reduced IL-10 serum levels were associated with a more unfavorable prognosis in patients with acute coronary syndromes [31]. IL-10 was an early outcome predictor in traumatic brain injury patients [32]. The differences in these studies might be caused by differences in the number of patients, time of onset of the disease, population, testing methods, and reagents. Additional large sample and multicenter studies assessing the role of IL-10 in ischemic stroke are warranted.

Welsh et al. [33] found that baseline circulating levels of IL-10 were positively associated with the risk of cardiovascular events among the elderly without prior cardiovascular events. Another study showed that the elevated serum IL-10 levels were related to the presence of depressive mood in patients with cardiovascular risk factors [34]. Dziedzic et al. [35] demonstrated that increased serum levels of IL-10 were significantly correlated with the Glasgow Coma Scale score in intracerebral hemorrhage patients. In addition, the temporospatial expression of IL-10 in the rat brain may contribute to worse outcomes [36]. The IL-10 level in serum did not show any significant correlation with the NIHSS score at the time of admission [37], which was not confirmed in our study.

The neuroprotective role of IL-10 in an experimental mouse stroke model had been verified [38]. Administration of hematopoietic cytokines could increase the expression of IL-10, which might provide a favorable microenvironment for neurogenesis after ischemic stroke [39]. A meta-analysis study showed that reduced serum levels of IL-10 might be a major player in the development and progression of cerebral infarction [40]; another cross-sectional study found that the lower serum IL-10 concentration and its selected genetic variations were significantly associated with an increased likelihood of ischemic stroke [41]. Serum levels of IL-10 in patients with unstable angina were significantly lower than those with chronic stable angina, suggesting that IL-10 has a protective role in atherosclerosis [42].

The elevated level of IL-10 in ischemic stroke patients might play a neuroprotective role through the following pathways: (1) the IL-10/STAT3 axis presents anti-inflammatory activities [43]. The use of anti-inflammatory medications might improve the prognosis following ischemic stroke. (2) IL-10 can play roles in limiting neuronal damage [43], increasing neuronal survival [44], and regulating adult neurogenesis [45]. IL-10 also can reduce the vulnerability of neurons to CNS ischemia and trauma [46]. (3) IL-10 could promote the activity of M2 macrophages in adipose tissue [47] or act directly on adipocytes to decrease their inflammatory response [48]. Cell-based therapies using M2-like macrophages could be protective therapeutic strategies against stroke [49]. Li et al. [50] showed that IL-10 could regulate microglial phagocytosis and macrophage infiltration after intracerebral hemorrhage by regulating CD36. Adipocyte fatty acid-binding protein plays a role in the stroke prognosis [51]. (4) IL-10 could provide neuroprotection by acting via IL-10 receptor and PI3K/AKT and STAT3 signal transduction pathways [52].

The following limitations need to be considered: (1) single-center and small sample (*N* = 236) researches need further verification. More samples with subtle designs are warranted in the future. (2) This cohort observational study cannot draw causal conclusions. (3) Blood samples were collected one time after admission. We could not obtain the change in serum IL-10 concentration after the onset of stroke patients. (4) We only tested serum levels of IL-10; other members of the IL-10 cytokine family, such as IL-19, IL-20, IL-22, and IL-24 [53], were not assessed. Thus, the relationship between IL-10, IL-10 cytokine family, and stroke prognosis could not be performed. (5) A meta-analysis indicated that IL-10-1082 A/G polymorphism was associated with ischemic stroke susceptibility in Asians [54]. However, in this study, the genetic polymorphism of IL-10 was not texted. Thus, the role of genetic polymorphism of IL-10 in stroke prognosis could not be confirmed. (6) IL-10 plays an important role in the cardiovascular system, and the blood, digestion, and especially diseases of the cardiovascular system are closely related [55]. However, we did not obtain that information in our study. Thus, the influence of other factors on IL-10 and stroke prognosis could not be excluded.

## 5. Conclusions

Reduced serum levels of IL-10 were independently associated with both the clinical severity at admission and a poor functional prognosis in ischemic stroke patients, suggesting that the anti-inflammatory cytokine IL-10 was an important prognostic determinant. Further studies are warranted to confirm whether the protective association of IL-10 with prognosis represents a causal pathway involved in the pathogenesis or the predictive effect of poor prognosis on ischemic stroke patients. This study may open up new strategies for the treatment and secondary prevention of stroke in the future.

## Figures and Tables

**Table 1:** Characteristics of the included patients.

Characteristics^†^	Patients with AIS
Participants	*N* = 236
Age (years)	
Median (IQR)	65 (56-76)
Mean age (SD)	65 (12.5)
Age groups	
<40	5 (2.1)
40-59	75 (31.8)
60-79	121 (51.3)
≥80	35 (14.8)
Sex	
Men	136 (57.6)
Women	100 (42.4)
Residence: urban	164 (69.5)
BMI (kg/m^2^)	
Mean (SD)	24.4 ± 3.9
Median (IQR)	24.2 (22.5-25.7)
Ethnicity	
Minority	11 (4.7)
Han	225 (95.3)
Education: college and above	35 (14.8)
Medical insurance	228 (96.6)
Marital status: married	224 (94.9)
Vascular risk factors	
Smoking status	
Nonsmokers	202 (85.6)
Past smokers	4 (1.7)
Current smokers	30 (12.7)
Consumption of alcohol	33 (14.0)
Family history of stroke	35 (14.8)
History of TIA	28 (11.9)
Hypertension	175 (74.2)
Diabetes	68 (28.8)
Hyperlipidemia	63 (26.7)
Atrial fibrillation	24 (10.2)
Obesity^††^	27 (11.4)
Stroke severity, median NIHSS score (IQR)	7 (3-11)
DWI lesion size, median (mL) (IQR)	15.8 (7.2-28.7)
Median arterial pressure (mm Hg) (IQR)	
Systolic	159 (145-167)
Diastolic	92 (80-106)
Median body temperature (°C) (IQR)	37.0 (36.5-37.5)
Stroke causative factors, *n* (%)	
Large-vessel occlusive	69 (29.2)
Small-vessel occlusive	55 (23.3)
Cardioembolic	42 (17.8)
Other	18 (7.6)
Unknown	52 (22.0)
Therapies before admission, *n* (%)	
Antihypertensive	142 (60.2)
Hypoglycemic	53 (22.5)
Anticoagulant	25 (10.6)
Statins	55 (23.3)
Acute treatment, *n* (%)	
IV thrombolysis	28 (11.9)
Mechanical thrombectomy	5 (2.1)
Mechanical thrombectomy and/or IV thrombolysis	31 (13.3)
Laboratory findings, median (IQR)	
Serum IL-10 level (pg/mL)	3.51 (2.61-5.24)
Serum IL-6 level (ng/mL)	16.1 (12.1-20.8)
Serum Hs-CRP (mg/dL)	0.82 (0.24-1.48)
Serum glucose level (mmol/L)	5.96 (5.15-6.77)
Triglycerides (mmol/L)	1.44 (1.14-1.89)
Total cholesterol (mmol/L)	4.13 (3.36-5.02)
HDL (mmol/L)	1.31 (1.08-1.62)
LDL (mmol/L)	2.10 (1.38-2.78)
Hospital stays, median days (IQR)	12 (9-17)
Hospital costs, median CNY (IQR)	9988 (8315-14153)
mRS at discharge, median (IQR)	2 (0-3)

^†^The results were presented as *n* (percentages) for categorical variables and as mean (standard deviation (SD)) for continuous variables. ^††^Obesity was defined as BMI ≥ 28.0 kg/m^2^. BMI: body mass index; CNY: Chinese Yuan Renminbi; TIA: transient ischemic attack; IQR: interquartile range; SD: standard deviation; NIHSS: National Institutes of Health Stroke Scale; DWI: diffusion-weighted imaging; mRS: modified Rankin Scale; IL-6: Interleukin-6; IL-10: Interleukin-10; Hs-CRP: hypersensitive-c-reactive-protein; LDL: high-density lipoprotein; LDL: low-density lipoprotein.

**Table 2:** Correlation analysis between IL-10 and different variables.

Variables	*r* (Spearman)	*P*
Age	0.052	0.425
Sex	-0.081	0.215
NIHSS	-0.221	0.001
Infarct size	-0.142	0.033
IL-6	-0.166	0.012
Hs-CRP	-0.108	0.103
Serum glucose	0.067	0.315
Stroke causative factors	0.134	0.044

NIHSS: National Institutes of Health Stroke Scale; IL-6: Interleukin-6; IL-10: Interleukin-10; Hs-CRP: hypersensitive-c-reactive-protein.

**Table 3:** Multivariate analysis of predictors of poor functional outcome (Rankin 3–6) and mortality^†^.

Variables	Poor functional outcome			Mortality		
	OR	95% CI	*P*	OR	95% CI	*P*
Age (increase per unit)	1.07	1.02-1.14	0.002	1.08	1.02-1.15	<0.001
Stroke severity, NIHSS > 6	15.14	3.03-30.11	<0.001	10.15	2.25-22.32	0.009
Acute stroke treatment	0.50	0.20-0.85	0.005	0.26	0.12-0.61	<0.001
IL-10 > 5.24 pg/mL^‡^	0.35	0.19-0.63	<0.001	0.24	0.11-0.52	<0.001
IL-6 (increase per unit)	1.13	1.04-1.21	0.003	1.16	1.02-1.25	0.002
Obesity (BMI ≥ 28 kg/m^2^)	1.85	1.05-2.82	0.032	2.05	1.07-4.05	0.183

^†^Multivariable model included the following variables: age, sex, obesity, vascular risk factors, therapies before admission, acute stroke treatment, stroke subtype, NIHSS at admission, infarct size, and serum levels of IL-6, Hs-CRP, and glucose. ^‡^IL-10 ≤ 5.24 pg/mL corresponding to the combination of the lowest three quartiles used as the reference. OR: odds ratio; CI: confidence interval; NIHSS: National Institutes of Health Stroke Scale; IL-6: Interleukin-6; IL-10: Interleukin-10; Hs-CRP: hypersensitive-c-reactive-protein.

## Data Availability

Please contact the corresponding author (Dr. Nan) for data requirements.
